# A Comparative In Vivo Analysis of Microhardness in Three Provisional Restorative Materials

**DOI:** 10.7759/cureus.96336

**Published:** 2025-11-07

**Authors:** Samanvitha Yerra, Sai Ram Challa, Priya Darshini Reddy, Rajendra P Bitragunta, Swetha Jujjavarapu, Lagadapati Sri Charitha, Raveen T Prathuri, Seema Gupta

**Affiliations:** 1 Department of Prosthodontics, Ganni Subba Lakshmi Dental College and Hospital, Rajahmundry, IND; 2 Department of Orthodontics, Kothiwal Dental College and Research Centre, Moradabad, IND

**Keywords:** computer-aided design, computer-aided manufacturing, microhardness, polymethyl methacrylate, provisional restorations

## Abstract

Introduction: Provisional restorations are vital in fixed prosthodontics, offering interim esthetics, function, and support until the final restorations are placed. This in vivo study compared the microhardness of three provisional restorative materials, namely, computer-aided design/computer-aided manufacturing (CAD/CAM)-milled polymethyl methacrylate (PMMA), bisacrylic (Protemp 4), and heat-cured PMMA, after 14 days of intraoral exposure.

Materials and methods: Thirty patients aged 18-50 years, requiring metal-ceramic crowns on the mandibular first or second molars, were selected from the Department of Prosthodontics, Ganni Subba Lakshmi Dental College and Hospital, Rajahmundry, India. After tooth preparation, impressions were made using irreversible hydrocolloids, and the casts were poured with die stones. Two temporary crowns per material were fabricated for each patient using the indirect impression technique: CAD/CAM-milled PMMA via intraoral scanning and milling, bisacrylic via putty index, and heat-cured PMMA via compression molding. One crown per pair (Group A, control) was tested immediately, whereas the other (Group B, case) was cemented intraorally with zinc oxide-eugenol for 14 days. All crowns were embedded in standardized wax blocks and subjected to Vickers microhardness testing (1 kg load, 15 s).

Results: Independent t-tests revealed a highly significant reduction in microhardness (p < 0.001) in the case groups compared with the control groups for all materials. Mean microhardness in the control group was 22.4 ± 1.2 kg/mm² for CAD/CAM-milled PMMA, 20.8 ± 1.0 kg/mm² for Protemp 4, and 25.6 ± 1.4 kg/mm² for heat-cured PMMA; in case groups, values dropped to 18.1 ± 0.9 kg/mm², 16.5 ± 0.8 kg/mm², and 22.3 ± 1.1 kg/mm², respectively. Heat-cured PMMA exhibited a significantly higher microhardness in both groups (p < 0.001), with CAD/CAM-milled PMMA and Protemp 4 showing comparable values.

Conclusion: The findings indicated that heat-cured PMMA offered superior resistance to intraoral degradation, guiding clinicians toward optimal material selection for durable provisional restorations. Further studies should assess their long-term performance and additional properties.

## Introduction

Provisional restorations play a pivotal role in fixed prosthodontics, serving as interim solutions that provide esthetics, function, and stabilization during the transition to definitive prostheses. These restorations are essential in various clinical scenarios, including full mouth rehabilitation, temporomandibular joint (TMJ) dysfunction therapies, and cases requiring extended provisional phases [1]. The choice of material for provisional restorations is critical because it must withstand the mechanical and biological challenges of the oral environment while maintaining structural integrity, esthetic appeal, and patient comfort [2]. Key mechanical properties such as microhardness, fracture resistance, and wear resistance significantly influence the longevity and performance of these restorations. Therefore, understanding how these properties are affected by in vivo conditions is vital for optimizing material selection based on specific clinical demands [3,4].

Polymethyl methacrylate (PMMA) and bisacrylic composites are among the most commonly used materials for provisional restorations because of their favorable handling characteristics, esthetics, and mechanical properties [1,2,5]. Advances in digital dentistry have introduced computer-aided design/computer-aided manufacturing (CAD/CAM) milling of PMMA blanks, offering improved precision and consistency compared with traditional fabrication methods [6]. Heat-cured PMMA and bisacrylic materials, such as Protemp, are widely used owing to their ease of fabrication and cost-effectiveness [7]. However, the performance of these materials in an oral environment, particularly over extended periods, remains underexplored. In vitro studies have provided valuable insights into the mechanical properties of these materials; however, they cannot fully replicate the complex biological and mechanical interactions encountered in vivo, such as salivary exposure, occlusal forces, and thermal cycling [3,7].

This study aimed to compare the microhardness of three provisional restorative materials, CAD/CAM-milled PMMA, bisacrylic (Protemp 4), and heat-cured PMMA, after 14 days of intraoral exposure. By evaluating microhardness changes in vivo, this study seeks to provide evidence-based insights into the durability and suitability of these materials for provisional restorations. Such findings are crucial for clinicians to make informed decisions and ensure optimal outcomes in prosthodontic treatment.

## Materials and methods

Study design

This prospective comparative in vivo study was conducted at the Department of Prosthodontics, Ganni Subba Lakshmi Dental College and Hospital, Rajahmundry, India, over a period of 12 months, from January 2023 to December 2023. Ethical approval was obtained from the Institutional Ethical Committee prior to the commencement of the study, ensuring compliance with the Declaration of Helsinki guidelines for human research. Written informed consent was obtained from all patients after providing detailed explanations of the study objectives, procedures, potential risks, and benefits, with assurance of confidentiality and the right to withdraw at any time without prejudice.

Patient eligibility

Patients were selected based on specific inclusion and exclusion criteria to ensure homogeneity and to minimize confounding factors. Inclusion criteria included patients aged 18 to 50 years, requiring metal-ceramic crowns on the mandibular first or second molars, healthy periodontium, and no contraindications to the procedures. Exclusion criteria included patients with parafunctional habits, such as bruxism, severely compromised teeth, systemic illnesses that could affect oral health or healing, poor oral hygiene, or periodontally weakened teeth. To minimize patient-related confounders, patients received standardized dietary guidelines restricting acidic (pH < 5.5), abrasive, or staining foods and beverages, with compliance tracked via daily food diaries. Oral hygiene was standardized using identical care kits (soft-bristle toothbrush and fluoride toothpaste), and a mid-study hygiene visit was conducted to reinforce compliance.

Sample size estimation

The sample size was estimated using the Gpower software (version 3.1.9.7; Heinrich-Heine-Universität Düsseldorf, Düsseldorf, Germany). Considering the 80% power and 5% alpha error for the study, a minimum sample size of 60 (10 per group) was required, with an effect size of 0.48. The effect size was obtained from a previous study that estimated the mean difference in microhardness between provisional crowns made using heat-cured PMMA and Protemp material [8].

The sample size per group required to detect a difference in means is given by: \begin{equation} n = \frac{2 (Z_{1-\alpha/2} + Z_{1-\beta})^2 \sigma^2}{\Delta^2} \end{equation}.

Where:

n is the sample size needed per group.

Z-value for the chosen significance level (α). For α = 0.05, this is 1.96.

Z-value for the desired power (1-β). For 80% power, this is 0.84; for 90%, it is 1.28.

sigma^2^ is the estimated variance (standard deviation squared) of the outcome in the population.

 delta^2^ is the clinically important difference in means.

Thus, 30 eligible patients were enrolled from the outpatient department, with two crown samples fabricated from each (resulting in 60 total samples). These were allocated into two main study groups: Group A (control, n = 30) and Group B (case, n = 30). Within each group, the 30 samples were further subdivided into three subsets of 10 based on the crown material type.

Methodology

The methodology began with patient selection and tooth preparation for metal-ceramic crown placement. Impressions were made using irreversible hydrocolloid impression material (Jeltrate, Dentsply Sirona, North Carolina, USA), followed by pouring master casts with die stone (Jade Stone, Whip Mix Corp., Kentucky, USA), and opposing casts with dental stone (Castone, Ransom & Randolph, Ohio, USA). The casts were mounted on a Hanau articulator (Hanau Wide-Vue, Whip Mix Corp., Kentucky, USA) using facebow transfer and centric relation records with protrusive records for programming condylar guidance. For each patient, two temporary crowns were fabricated per group using the indirect over-impression technique, with one designated as the control (Group A, tested immediately after fabrication) and the other as the case (Group B, cemented intraorally for 14 days). Necessary occlusal adjustments were made to ensure proper function and patient comfort.

The samples were divided into three groups based on the provisional material used. Group 1 used CAD/CAM-milled PMMA, in which mounted casts were scanned using an intraoral scanner (Cerec Primescan, Dentsply Sirona, North Carolina, USA) to capture detailed images of the prepared tooth and occlusion. The scans were processed on a computer-aided design platform, exported in stereolithography (STL) format, and milled from PMMA blanks (Primotec USA, Connecticut, USA) using an inLab MC X5 milling machine (Dentsply Sirona, North Carolina, USA). Group 2 used bisacrylic material (Protemp 4, 3M Company, Minnesota, USA), where wax patterns were fabricated on the prepared tooth using Type II inlay wax (Corning Inlay Wax, Keystone Industries, New Jersey, USA) after applying a separating medium, carved with a PKT kit (P.K. Thomas waxing instruments, HuFriedy Group, Illinois, USA), and polished. A putty index was created from the wax pattern, and the bisacrylic material, dispensed from cartridges with base and catalyst mixed via auto-mixing tips, was loaded into the index for crown fabrication, followed by finishing and polishing. This process was repeated for the twin crown. Group 3 used heat-cured PMMA (DPI Heat Cure, Bombay Burmah Trading Corp. Ltd., Maharashtra, India), followed by wax pattern fabrication similar to Group 2, but using a compression molding technique to process the heat-cure resin from the patterns, with subsequent trimming and polishing.

**Figure 1:**
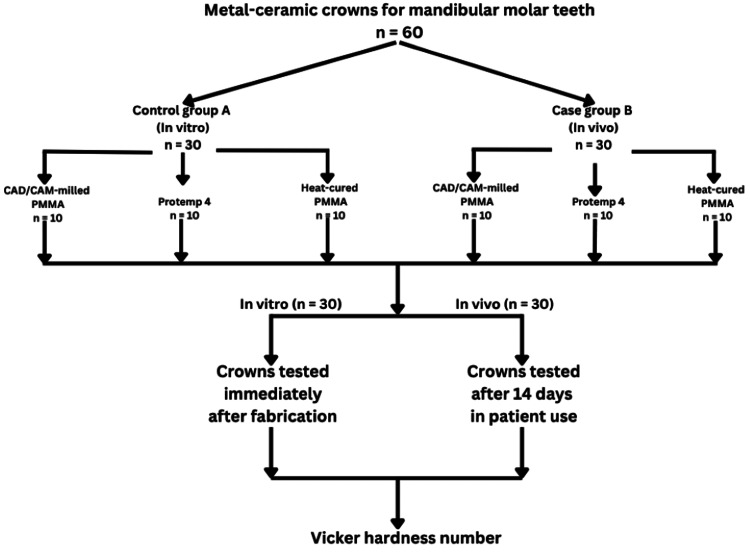
Study flowchart. CAD/CAM: computer-aided design/computer-aided manufacturing, PMMA: polymethyl methacrylate Created by the authors using Canva (www.canva.com)

For all case groups (Group B), the crowns were cemented intraorally using zinc oxide-eugenol cement (Temp-Bond, Kerr Corp., California, USA) for 14 days. After 14 days, the crowns were retrieved, rinsed in distilled water, air-dried for 10 minutes, and stored in sealed containers at room temperature for testing within 24 hours. The control crowns (Group A) were tested within one hour of fabrication in a climate-controlled laboratory (220C, 50% humidity). All crowns were embedded in standardized silicone-molded wax blocks (20 × 20 × 10 mm). Subsequently, the permanent restorations were placed. To differentiate the groups, acrylic color coatings were applied: purple for CAD/CAM-milled PMMA group, self-cure pink for bisacrylic group, and clear for heat-cured PMMA group. The samples underwent Vickers microhardness testing, in which a load of 1 kg was applied to the crown surface for 15 seconds using a Vickers hardness tester (HMV-G21, Shimadzu Corp., Kyoto, Japan), and the diagonal lengths of the indentations were measured under a microscope to calculate the microhardness values in kg/mm² (Figure 1).

Prior to testing, the Vickers hardness tester was calibrated using standard reference blocks according to the manufacturer’s guidelines to ensure an accuracy within ±2% tolerance. Reliability was assessed through intra-examiner calibration, in which the principal investigator performed repeated measurements on 10 pilot samples, yielding an intraclass correlation coefficient of 0.92, indicating high reproducibility. Blinding was maintained during microhardness measurements to minimize bias, with an independent assessor evaluating the indentations without knowledge of the group assignments.

Statistical analysis

Statistical analyses were performed using IBM SPSS Statistics for Windows, Version 25.0 (released 2017, IBM Corp., Armonk, NY). The normality of the data was confirmed using the Shapiro-Wilk test. An independent samples t-test was used to compare microhardness between the groups. One-way analysis of variance (ANOVA) was used for comparisons within groups, followed by Tukey’s post-hoc test for pairwise comparisons between materials. Statistical significance was set at p < 0.05, and a p-value of < 0.001 was considered highly statistically significant.

## Results

The demographic and baseline characteristics of the study participants across the three groups are presented in Table 1. Statistical analysis revealed no significant differences between groups for any of the recorded variables. The distribution of sex (p = 0.626), mean age (p = 0.811), proportion of control to case groups (p = 0.328), and type of mandibular molar tooth treated (p = 0.561) were all comparable.

**Table 1:** Demographic characteristics of the sample. p > 0.05: non-significant, test statistics for sex, group and mandibular molar tooth was calculated by chi-square test, test statistics for age was calculated by one-way analysis of variance (ANOVA) test. Categorical variables (sex, groups, and mandibular molar tooth) are presented as frequency (n) and percentage (%), and continuous variable (age) is presented as mean and standard deviation (SD). CAD/CAM: computer-aided design/computer-aided manufacturing, PMMA: polymethyl methacrylate

Variables	Expressed as	CAD/CAM-milled PMMA	Protemp 4	Heat-cured PMMA	Test statistics	p-value
Sex	Male n (%)	8 (40%)	10 (50%)	8 (40%)	2.23	0.626
	Female n (%)	12 (60%)	10 (50%)	12 (60%)		
Age	Years (mean ± SD)	34.42 ± 4.56	32.60 ± 5.25	34.92 ± 5.12	0.78	0.811
Groups	In vitro (Control Group A)	10 (50%)	10 (50%)	10 (50%)	1.56	0.328
	In vivo (Case Group B)	10 (50%)	10 (50%)	10 (50%)		
Mandibular molar tooth	First molar	11 (55%)	14 (70%)	12 (60%)	2.37	0.561
	Second molar	9 (45%)	6 (30%)	8 (40%)		

Independent t-test analysis revealed a highly statistically significant reduction (p < 0.001) in the microhardness of all three provisional materials after 14 days of intraoral cementation (Case Group B) compared to their pre-cementation state (Control Group A). The mean microhardness values for the CAD/CAM-milled PMMA, Protemp 4, and heat-cured PMMA were consistently lower in Group B. This significant decrease in microhardness across all materials indicated that the intraoral environment and functional loading during the 14-day period substantially compromised the surface integrity of the provisional restorations, potentially affecting their clinical durability and protective function (Table 2).

**Table 2:** Comparison of microhardness in Vickers hardness number (VHN), expressed as kg/mm² (kilograms per square millimeter) between groups based on material property. Control Group A: provisional crowns were fabricated and tested immediately within 24 hours. Case Group B: provisional crowns were fabricated and cemented for 14 days intraorally, then retrieved and tested. *p < 0.001 denotes highly statistically significant values using with independent t-test, and microhardness values are expressed as mean and standard deviation (SD). CAD/CAM: computer-aided design/computer-aided manufacturing, PMMA: polymethyl methacrylate

Material type	Control Group A (Mean ± SD)	Case Group B (Mean ± SD)	t statistics	p-value
CAD/CAM-milled PMMA	16.92 ± 1.10	13.94 ± 0.78	9.699	0.001*
Protemp 4	16.98 ± 0.50	14.11 ± 0.53	187.886	0.001*
Heat-cured PMMA	22.03 ± 1.70	20.89 ± 1.74	22.851	0.001*

A highly statistically significant difference in microhardness was observed among the three materials in Control Group A (p < 0.001) (Table 3).

**Table 3:** Comparison of microhardness in Vickers hardness number (VHN), expressed as kg/mm² (kilograms per square millimeter) of different material types within Control Group A. Control Group A: provisional crowns were fabricated and tested immediately within 24 hours, *p < 0.001 denotes highly statistically significant values using one-way analysis of variance (ANOVA), and microhardness values are expressed as mean and standard deviation (SD). PMMA: polymethyl methacrylate, CAD/CAM: computer-aided design/computer-aided manufacturing

Material type	Mean ± SD	F value	p-value
CAD/CAM-milled PMMA	16.92 ± 1.10	53.653	0.001*
Protemp 4	16.98 ± 0.50		
Heat-cured PMMA	22.03 ± 1.70		

Post-hoc analysis revealed that heat-cured PMMA exhibited significantly higher microhardness values than both CAD/CAM-milled PMMA and Protemp 4, which were statistically similar. The material composition significantly influences the initial microhardness (Table 4).

**Table 4:** Post-hoc analysis for Control Group A. *p < 0.001 denotes highly statistically significant values post-hoc analysis, and microhardness values are expressed as mean and standard deviation (SD). PMMA: polymethyl methacrylate, CAD/CAM: computer-aided design/computer-aided manufacturing

Post-hoc test	Mean difference	t statistics	p-value
CAD/CAM-milled PMMA vs. heat-cured PMMA	-5.11	-9.024	0.001*
Protemp 4 vs. CAD/CAM-milled PMMA	0.06	-0.106	0.994
Heat-cured PMMA vs. Protemp 4	5.05	8.918	0.001*

A highly statistically significant difference in microhardness was found among the materials in Case Group B (p < 0.001) (Table 5).

**Table 5:** Comparison of microhardness in Vickers hardness number (VHN), expressed as kg/mm² (kilograms per square millimeter) of different material types within Case Group B. Case Group B: provisional crowns were fabricated and cemented for 14 days intraorally, then retrieved and tested, *p < 0.001 denotes highly statistically significant values using one-way analysis of variance (ANOVA), and microhardness values are expressed as mean and standard deviation (SD). CAD/CAM: computer-aided design/computer-aided manufacturing, PMMA: polymethyl methacrylate

Material type	Mean ± SD	F-value	p-value
CAD/CAM-milled PMMA	13.94 ± 0.78	120.069	0.001*
Protemp 4	14.11 ± 0.53		
Heat-cured PMMA	20.89 ± 1.74		

Post-hoc analysis confirmed that heat-cured PMMA maintained significantly higher microhardness after 14 days intraorally compared to CAD/CAM-milled PMMA and Protemp 4, which showed similar lower values. The material composition dictated the durability in the oral environment (Table 6).

**Table 6:** Post-hoc analysis for Case Group B. *p < 0.001 denotes highly statistically significant values post-hoc analysis, and microhardness values are expressed as mean and standard deviation (SD). CAD/CAM: computer-aided design/computer-aided manufacturing, PMMA: polymethyl methacrylate

Post-hoc test	Mean difference	t statistics	p-value
CAD/CAM-milled PMMA vs. heat-cured PMMA	-6.95	-13.583	0.001*
Protemp 4 vs. CAD/CAM-milled PMMA	0.17	-0.332	0.941
Heat-cured PMMA vs. Protemp 4	6.78	13.251	0.001*

## Discussion

The observed reduction in microhardness across all tested provisional materials after intraoral exposure underscores the degradative influence of the oral environment on the polymeric restorations. This decline can be explained by multifaceted interactions within the mouth, including constant exposure to saliva, which promotes water sorption and hydrolysis of ester bonds in the resin matrix, leading to chain scission and surface softening [9]. The water that is absorbed migrates and infiltrates the interstitial regions between polymeric chains, thereby inducing their separation and resulting in three-dimensional expansion [10]. A correlation has been documented between the volume of absorbed water and its detrimental impact on physical characteristics [11].

Mastication further imposes repetitive mechanical stress, causing micro-abrasive wear and subsurface fatigue cracks that exacerbate the indentation susceptibility. Furthermore, thermocycling from ingested foods and beverages induces dimensional changes, fostering internal stress and microcrack propagation [12]. These processes collectively diminish the resistance of the material to deformation, as demonstrated in previous in vitro investigations simulating oral conditions through solvent immersion and thermocycling, where significant microhardness losses were noted in both methacrylate and bisacryl resins [12,13]. These findings align with the current in vivo results; however, the real-time functional loading and biological factors (such as enzymatic activity) likely intensify the effect, highlighting why provisional restorations may fail prematurely if not selected judiciously for the anticipated service duration.

The superior initial microhardness of heat-cured PMMA over CAD/CAM-milled PMMA and bisacryl (Protemp 4) can be attributed to its fabrication process, which involves high-temperature polymerization under pressure, resulting in more complete monomer conversion, higher molecular weight, and denser cross-linked network. This enhances the ability of the material to resist plastic deformation under loading. In comparison, bisacrylic composites such as Protemp 4 rely on self-polymerization, which often leaves higher residual monomers and a less homogeneous matrix, making them more vulnerable to indentation, despite their bifunctional dimethacrylate structure that provides initial rigidity [8]. CAD/CAM-milled PMMA, derived from industrially pre-polymerized blocks, offers precision but may incorporate additives or have altered chain architectures that reduce hardness relative to lab-processed heat-cured variants [14]. These compositional and processing differences are supported by comparative studies, where heat-polymerized methacrylates have shown enhanced mechanical properties owing to their minimized porosity and improved polymerization efficiency [1].

Digholkar et al. [15] conducted an in vitro study, where they compared the flexural strength of heat-cured PMMA and CAD/CAM-milled PMMA-fabricated provisional restorations, and found that there was no significant difference between the flexural strength values. Mehraj et al. [8] compared the microhardness of heat-cured PMMA and Protemp 4 and concluded that both had no statistically significant difference in microhardness. Hada et al. [16] compared the flexural strength and water sorption of CAD/CAM-milled PMMA and heat-cured PMMA discs, and found that the flexural strength of CAD/CAM-milled PMMA was significantly higher than that of heat-cured PMMA discs, whereas no significant differences were observed for water sorption for 24 h. Similar results were reported by Wechkunanukul et al. [17], who reported higher microhardness values for CAD/CAM-milled PMMA. The difference in results could be attributed to the fact that these studies were in vitro.

However, conflicting reports indicate that bisacrylic materials occasionally outperform auto-polymerized PMMA in terms of microhardness, possibly owing to brand variations or testing protocols, although heat-cured PMMA consistently excels in scenarios requiring robust initial strength. The similarity between CAD/CAM-milled PMMA and bisacrylic material (Protemp 4) in this study echoes evaluations where milled or rapid-prototyped materials exhibit comparable surface properties, attributed to their shared reliance on preformed polymers rather than in-lab curing [8,17].

After exposure, the sustained advantage of heat-cured PMMA suggests greater resilience to oral stressors. Its hydrophobic characteristics and stable polymer chains likely limit fluid penetration and hydrolytic breakdown, preserving structural integrity better than the more hydrophilic bisacryl, which readily absorbs water, leading to swelling and reduced hardness [1,15]. The CAD/CAM-milled PMMA, while benefiting from uniform milling, may experience similar sorption issues if the block formulation includes hydrophilic components. Yıldırım et al. [18] reported that after thermocycling, CAD/CAM-milled PMMA and heat-cured PMMA showed higher fracture strength and surface microhardness than the bisacrylic material (Protemp 4). The in vivo setting of this research amplifies these insights, as patient-specific factors such as occlusal dynamics accelerate wear beyond artificial simulations, explaining why heat-cured materials maintain superiority in functional contexts.

These material-specific behaviors emphasize the role of the polymerization method and chemistry in dictating performance. While in vitro tests provide foundational data, the current in vivo approach reveals nuanced interactions, such as how the esthetic advantages of bisacrylic may trade off against durability in moist environments. This contributes to the evolving understanding that provisional materials must balance handling, esthetics, and mechanics, with heat-cure options offering an edge in demanding cases.

Clinicians should prioritize heat-cured PMMA for prolonged provisional phases, such as full-mouth rehabilitation or TMJ treatments, to minimize the risks of surface wear, marginal leakage, or abutment damage from softened restorations. For routine short-term use, CAD/CAM-milled PMMA or bisacrylic material (Protemp 4) provides efficient alternatives with adequate initial properties, but routine checks for degradation are advisable. Incorporating surface sealants or optimizing occlusion could further enhance longevity and improve patient outcome and treatment predictability.

Limitations

The constraints of this study encompassed a 14-day assessment duration, which, although pertinent for provisional application, may not accurately depict performance over prolonged usage exceeding two weeks, thereby potentially undervaluing the cumulative deterioration. The sample of 30 patients, focused on posterior molars, limited extrapolation to anterior teeth or diverse populations, including those with varying dietary habits or systemic conditions. Despite methodological controls, residual interpatient variability (such as salivary flow) could influence the results. Future studies should extend the duration, incorporate multicenter designs, and assess complementary properties, such as fracture toughness, for comprehensive insights.

## Conclusions

This in vivo study demonstrated that the intraoral environment significantly compromised the microhardness of the CAD/CAM-milled PMMA, bisacrylic (Protemp 4), and heat-cured PMMA provisional restorations after 14 days of functional use. Among the tested materials, heat-cured PMMA exhibited superior initial and post-exposure microhardness, indicating a greater resistance to degradation under oral conditions. These findings suggest that material composition and polymerization techniques critically influence the durability of provisional restorations, with heat-cured PMMA being the most resilient. These results provide valuable insights for clinicians in selecting provisional materials tailored to clinical demands, particularly in cases requiring extended interim restorations. Future research should explore longer exposure periods and additional mechanical properties to guide material selection in prosthodontics.
